# Insights on epidemiology, morbidity and mortality of Cushing’s disease in Northern Ireland

**DOI:** 10.1530/ERC-24-0028

**Published:** 2024-07-08

**Authors:** Paul Benjamin Loughrey, Brian Herron, Stephen Cooke, Philip Weir, Jayna Elizabeth Smyth, Karen R Mullan, Estelle G Healy, Jane Evanson, Stephanie G Craig, Jacqueline A James, Márta Korbonits, Steven J Hunter

**Affiliations:** 1 Patrick G Johnston Centre for Cancer Research, Queen’s University Belfast, Belfast, UK; 2 Regional Centre for Endocrinology and Diabetes, Belfast Health and Social Care Trust, Belfast, UK; 3 Department of Cellular Pathology, Belfast Health and Social Care Trust, Belfast, UK; 4 Department of Neurosurgery, Belfast Health and Social Care Trust, Belfast, UK; 5 Department of Radiology, Barts Health NHS Trust, London, UK; 6 Northern Ireland Biobank, Belfast Health and Social Care Trust, Belfast, UK; 7 Department of Endocrinology, William Harvey Research Institute, Barts and The London School of Medicine and Dentistry, Queen Mary University of London, London, UK

**Keywords:** corticotrophinoma, Cushing’s disease, epidemiology, neurosurgery, pituitary

## Abstract

Cushing’s disease is a rare condition that occurs due to an adrenocorticotrophin-producing corticotrophinoma arising from the pituitary gland. The consequent hypercortisolaemia results in multisystem morbidity and mortality. This study aims to report incidence, clinicopathological characteristics, remission outcomes and mortality in a regional pituitary neurosurgical cohort of patients diagnosed with Cushing’s disease in Northern Ireland (NI) from 2000 to 2019. Clinical, biochemical and radiological data from a cohort of patients operated for Cushing’s disease were retrospectively collected and analysed. Fifty-three patients were identified, resulting in an estimated annual incidence of Cushing’s disease of 1.39–1.57 per million population per year. Females accounted for 72% (38/53) of the cohort. The majority (74%, 39/53) of corticotrophinomas were microadenomas and in 44% (17/39) of these no tumour was identified on preoperative magnetic resonance imaging. Histopathological characterisation was similarly difficult, with no tumour being identified in the histopathological specimen in 40% (21/53) of cases. Immediate postoperative remission rates were 53% and 66% when considering serum morning cortisol cut-offs of ≤ 50 nmol/L (1.8 µg/dL) and ≤ 138 nmol/L (5 µg/dL), respectively, in the week following pituitary surgery. Approximately 70% (37/53) of patients achieved longer-term remission with a single pituitary surgery. Three patients had recurrent disease. Patients with Cushing’s disease had a significantly higher mortality rate compared to the NI general population (standardised mortality ratio 8.10, 95% CI 3.3–16.7, *P* < 0.001). Annual incidence of Cushing’s disease in NI is consistent with other Northern European cohorts. Functioning corticotrophinomas are a clinically, radiologically and histopathologically elusive disease with increased mortality compared to the general population.

## Introduction

Cushing’s disease (CD) is a rare condition of endogenous hypercortisolaemia resulting from an adrenocorticotrophin (ACTH)-secreting pituitary neuroendocrine tumour. Annual incidence of CD has been estimated at 1.2–1.8 cases per million population per year (Lindholm *et al.* 2001, Bolland *et al.* 2011, Agustsson *et al.* 2015, Ragnarsson *et al.* 2019, Wengander *et al.* 2019). Despite the almost invariably benign nature of corticotrophinomas, CD-associated hypercortisolaemia results in considerable comorbidity and mortality (Valassi *et al.* 2011, Limumpornpetch *et al.* 2022). Comorbidities include hypertension, type 2 diabetes mellitus, cardiovascular disease, cerebrovascular disease and opportunistic infections (Fleseriu *et al.* 2021, Valassi 2022). Mortality in CD has repeatedly been shown to be raised in relation to the general population and is largely due to cardiovascular events, and this persists despite cure of CD with bilateral adrenalectomy (Fountas *et al.* 2020, Limumpornpetch *et al.* 2022). Treatment of CD has evolved from first-line treatment with bilateral adrenalectomy and conventional radiotherapy to more contemporary approaches with trans-sphenoidal pituitary surgery as a first-line, potentially curative therapeutic intervention (Plotz *et al.* 1952, Fleseriu *et al.* 1952). Despite refinement of CD diagnostic techniques and therapeutic interventions, remission rates following trans-sphenoidal surgery are variable (Alexandraki *et al.* 2013, Brady *et al.* 2021, Catalino *et al.* 2023). Prognostication at an early stage of postoperative follow-up can be difficult; some patients with suspicion of disease persistence later remit without further intervention and others with initial apparent remission go on to develop disease recurrence (Atkinson *et al.* 2005).

The objective of this study was to report incidence, clinicopathological characteristics, trans-sphenoidal surgery remission outcomes and mortality in a regional pituitary neurosurgical cohort of adult patients diagnosed with CD.

## Methods

This is a retrospective population-based cohort study which involved no intervention beyond usual patient care. The search term ‘pituitary’, as per systemised nomenclature of medicine terms, was applied to the regional neuropathological laboratory information management system covering the entirety of Northern Ireland (NI) from 1 January 2000 to 31 December 2019. This search was cross referenced with a registered database held by the neurosurgical team within the Belfast Health and Social Care Trust to identify a cohort representative of all surgically managed CD in NI for the study period.

Clinicopathological parameters were extracted from multiple sources including electronic patient records and hospital notes before collation into a single retrospective audit database.

The clinical diagnosis of CD was made at the Regional Centre for Endocrinology and Diabetes at the Royal Victoria Hospital Belfast in accordance with local protocols which require at least two tests evidencing hypercortisolaemia. Diagnoses were confirmed using biochemical tests to include 24-h urinary free cortisol (UFC), 1 mg overnight dexamethasone suppression testing, plasma ACTH, serum cortisol, late-night salivary cortisol, corticotrophin-releasing hormone testing, bilateral inferior petrosal sinus sampling and historical low and high dose dexamethasone suppression testing. Imaging included computed tomography and magnetic resonance imaging of the pituitary. All patients were treated with trans-sphenoidal pituitary surgery as first-line therapy.

In the Royal Victoria Hospital, Belfast, oral hydrocortisone was continued postoperatively until 24 h before a morning serum cortisol which was measured in the first postoperative week. Decisions on further therapy were made based on this biochemical assessment, detailed clinical assessment and pre and postoperative imaging characteristics.

Patients were followed up longer term postoperatively by clinical and biochemical assessment at the supervising clinician’s discretion. Follow-up biochemical assessment included 24-h UFC, plasma ACTH measurements, 1 mg overnight dexamethasone suppression testing and serum cortisol testing where clinically indicated. Follow-up concluded at point of last clinical and biochemical assessment, geographic relocation outside of NI, or death.

Patients were initially categorised according to immediate post operative morning serum cortisol levels. Immediate remission was defined by a morning serum cortisol of ≤ 50 nmol/L (‘strict criteria’) in the week following pituitary surgery. Persistent disease was defined as a morning serum cortisol > 50 nmol/L in the week following pituitary surgery. Patients were then further defined during follow-up as long-term remission, persistent disease or recurrence. Long-term remission was defined as those patients who underwent pituitary surgery followed by ongoing absence of hypercortisolism until the point of last follow-up. Persistent disease was defined by a morning serum cortisol > 50 nmol/L in the week following pituitary surgery followed by ongoing evidence of hypercortisolism requiring further therapeutic intervention. Recurrence was defined by biochemical and clinical evidence of hypercortisolism having initially achieved a morning cortisol ≤ 50 nmol/L in the week following pituitary surgery followed by a period of hypo or eucortisolism during follow-up.

A cut-off of ≤ 138 nmol/L (‘standard criteria’) was also investigated to define immediate remission criteria as above to facilitate comparison with other cohorts and clinical guidelines.

### Ethical compliance

The anonymised data presented were collected retrospectively as part of an audit within the Belfast Health and Social Care Trust, registered with the audit and quality improvement department and assigned the approval number 6057.

### Statistical analysis

Statistical analyses were carried out with GraphPad Prism 10.1.0. Data are expressed as median and interquartile range, unless otherwise stated.

NI census data from 2001 and 2021 were used to obtain population estimates for NI. An estimated range for annual incidence of CD in NI was calculated by taking the total observed cases and dividing them by the number of years in the study observation period and by the NI population figures. Calendar year-, age- and sex-specific mortality figures were obtained from NI census data and used to estimate expected deaths. Standardised mortality ratios (SMRs) were calculated as a ratio of observed over expected deaths using the openepi.com SMR calculator (https://www.openepi.com/SMR/SMR.htm) and assessed using 95% CIs and Fisher’s exact test.

Categorical variables were compared using Chi-squared tests or Fisher’s exact tests as appropriate. *t*-tests were used to compare continuous variables. Recurrence free survival was estimated using Kaplan–Meier curves and the log-rank test used to evaluate for significant differences. A *P-*value < 0.05 was considered significant.

## Results

Fifty-three patients were diagnosed and received neurosurgical intervention as primary therapy for CD in NI between 1 January 2000 and 31 December 2019. All patients underwent trans-sphenoidal surgery as first-line therapy for CD. Demographic, radiological and histopathological details are summarised in Table 1. Eight patients with macroadenomas did not have bilateral inferior petrosal sinus sampling. The remainder of the cohort underwent bilateral inferior petrosal sinus sampling and all patients had a basal central to peripheral gradient of > 2 and corticotrophin-releasing hormone-stimulated central-to-peripheral gradient > 3.

**Table 1:** Summary of demographic, radiological and histopathological characteristics of patients operated for a new diagnosis of CD in NI 2000–2019 inclusive. Age and diagnostic delay data presented as median and interquartile range.

Patients operated for a new diagnosis of CD in NI 2000–2019	53
Age	41.0 (31.3–54.8)
Sex Female Male	38/53 (72%) 15/53 (28%)
Diagnostic delay^a^	3.0 (2.0–7.0)
Tumour size Macroadenoma Microadenoma Visible on MRI Not visible on MRI	14/53 (26%) 39/53 (74%) 22/39 (56%) 17/39 (44%)
Tumour extension/invasion^b^ Suprasellar Optic chiasm contact Sphenoid Knosp grade 0 1 2 3a 3b 4	9/44 (21%) 6/9 (67%) 3/44 (7%) 38/44 (86%) 3/44 (7%) 1/44 (2%) 2/44 (5%) 0/44 (0%) 0/44 (0%)
Histopathology ACTH immunopositive Prolactin immunopositive No tumour identified	30/53 (57%) 2/53 (4%) 21/53 (40%)

^a^Diagnostic delay data were available for 35/53 (66%) patients; ^b^MRI data were available for a direct retrospective review in 44/53 (83%) patients.

In 2001, NI had a population of 1,685,267 and in 2021, the population was 1,903,175 (NISRA 2001, 2021). Based on these census figures an annual incidence of 1.39–1.57 CD cases per million population per year is estimated (NISRA 2001, 2021).

At presentation, weight gain, headache and fatigue were most the most documented symptoms in 57% (30/53), 19% (10/53) and 19% (10/53) of the population respectively. Hirsutism (34%, 13/38) and menstrual irregularities (32%, 12/38) were the most documented presenting complaints for females. One patient had clinical and radiological findings consistent with apoplexy at diagnosis.

The cohort displayed a range of comorbidities, but documented formal diagnoses of hypertension (79%, 42/53); type 2 diabetes mellitus (23%, 12/53); cerebrovascular disease and psychiatric disorders (both 17%, 9/53); cardiovascular disease, osteoporosis, low-impact fracture (all 13%, 7/53); and venous thromboembolism (VTE) (9%, 5/53) were prevalent. Hypokalaemia prior to surgery was evident in approximately 34% (18/53) of the cohort. Preoperative 24-h UFC levels were available in 48 patients. In one patient the 24-h UFC was so high that it was outside the reported range and so was excluded from analysis. When considering peak 24-h UFC levels relative to the upper limit of the reference range, levels were significantly higher in patients with hypokalaemia detected preoperatively (8.7× vs 3.6× upper limit of reference range, *P* = 0.030).

In two patients, the data were not available to decide on the presence or absence of transient arginine vasopressin deficiency (AVP-D). Of the remaining 51 patients, 11 (22%) had transient AVP-D (duration < 72 h), which resolved. A further ten patients (19%) proceeded to develop permanent AVP-D. Of these ten patients, eight achieved immediate postoperative serum cortisol ≤ 50 nmol/L. Of the remaining two, one had a Knosp grade 3a macroadenoma and the other with a tumour not visible on MRI achieved cure with a repeat surgery (Micko *et al.* 2015). Patients with tumour not visible on MRI were not significantly more likely to have perioperative AVP-D or permanent AVP-D compared to those with a visible tumour < 10 mm in maximal diameter (*P* = 0.320). Most patients had a straightforward perioperative course, with 33/50 (three unknown data) experiencing no perioperative complications and 8/50 (16%) having a small cerebrospinal fluid leak that was repaired intraoperatively. More serious complications included presumed meningitis (3/50, 6%, causal organism not identified), persistent postoperative cerebrospinal fluid leak (5/50, 10%) and perioperative myocardial infarction (1/50, 2%). There were no perioperative deaths.

There was no significant difference in the likelihood of achieving strict immediate remission when comparing patients with tumour < 10 mm in maximal diameter visible on initial MRI to those without tumour visible on initial MRI (*P* = 0.716). No patients with ≥ grade 2 Knosp cavernous sinus invasion achieved strict immediate postoperative remission (Micko *et al.* 2015).

Two tumours were immunoreactive for prolactin (with the report stating entirely negative for ACTH) and these patients achieved postoperative remission without further therapeutic interventions. There was no significant difference in immediate remission rate in those patients who had identifiable corticotrophinoma with positive immunostain for ACTH in their histological sample compared to those who did not (*P* = 0.568).

**Figure 1:**
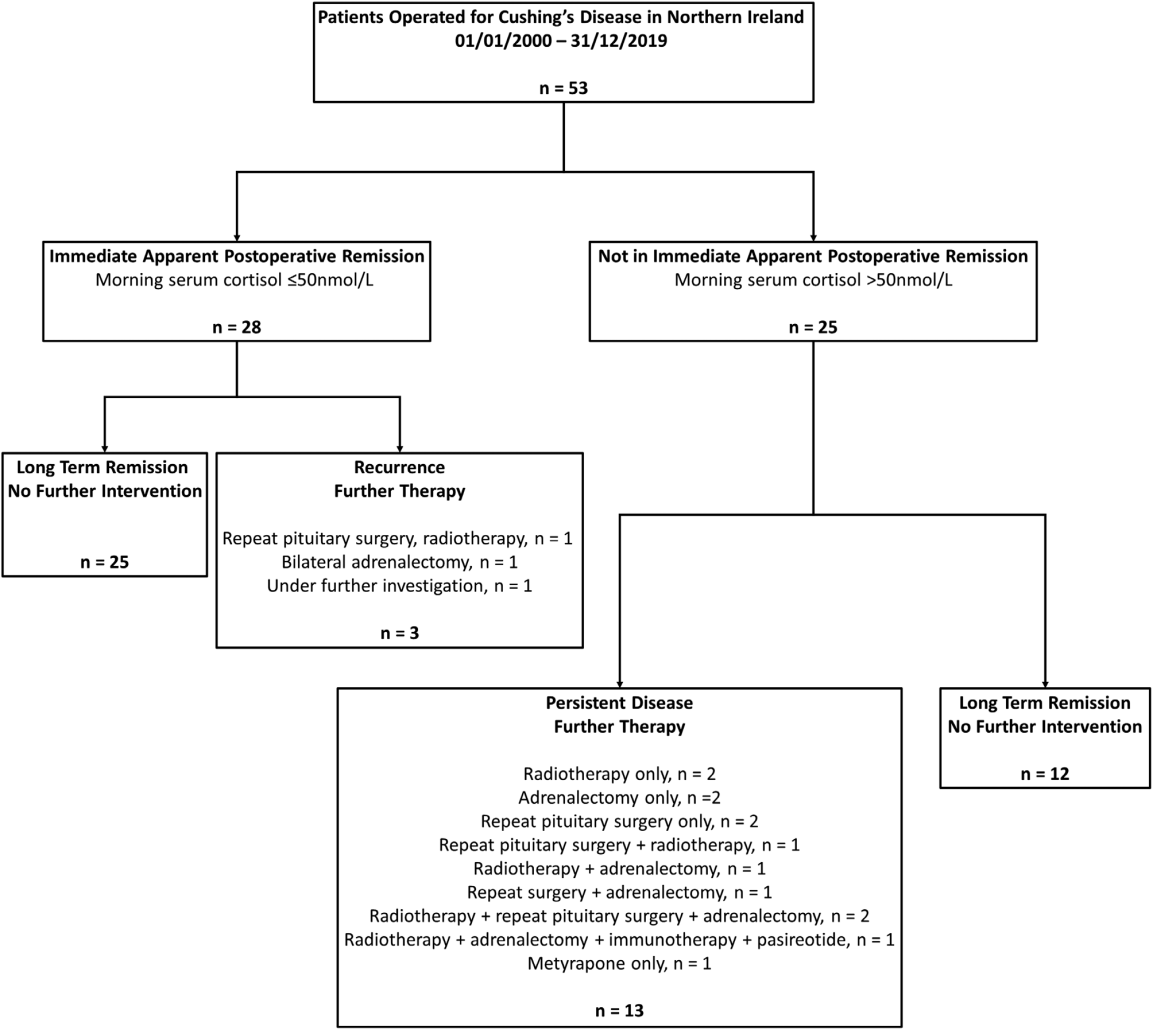
CD treatment outcomes in NI 01/01/2000-31/12/2019.

Eighteen of the cohort were treated with metyrapone preoperatively. When categorising according to immediate postoperative remission, 28/53 (53%) patients had morning serum cortisol ≤ 50 nmol/L (Fig. 1). Twelve patients who did not demonstrate strict immediate postoperative remission went on to longer-term remission without further therapeutic interventions, and nine of them received preoperative metyrapone therapy. When comparing the 24-h UFC levels of these patients with those who achieved strict immediate postoperative remission, there was no significant difference between the degrees of hypercortisolaemia preoperatively (*P* = 0.379). Thirty-five out of 53 (66%) patients met standard immediate postoperative remission criteria (≤ 138 nmol/L).

Ultimately, 70% of patients achieved long-term remission with a single pituitary surgery only (Fig. 1). The median follow-up of the cohort was 103.5 (85.3–154.2) months, totalling 539.6 person-years. Three patients had evidence of recurrent disease after meeting strict remission criteria, with a median time to recurrence of 5.6 years (Fig. 1).

At the close of the study, 51/53 (96%) patients had control of hypercortisolaemia; one patient had definite ongoing disease activity with aggressive disease despite optimal standard therapies (Raverot *et al.* 2018). The other patient was under investigation for recurrence 9.8 years postoperatively with a raised 24-h UFC cortisol which had previously been normal on a background of having achieved apparent strict immediate remission with postoperative morning serum cortisol 19 nmol/L.

**Figure 2:**
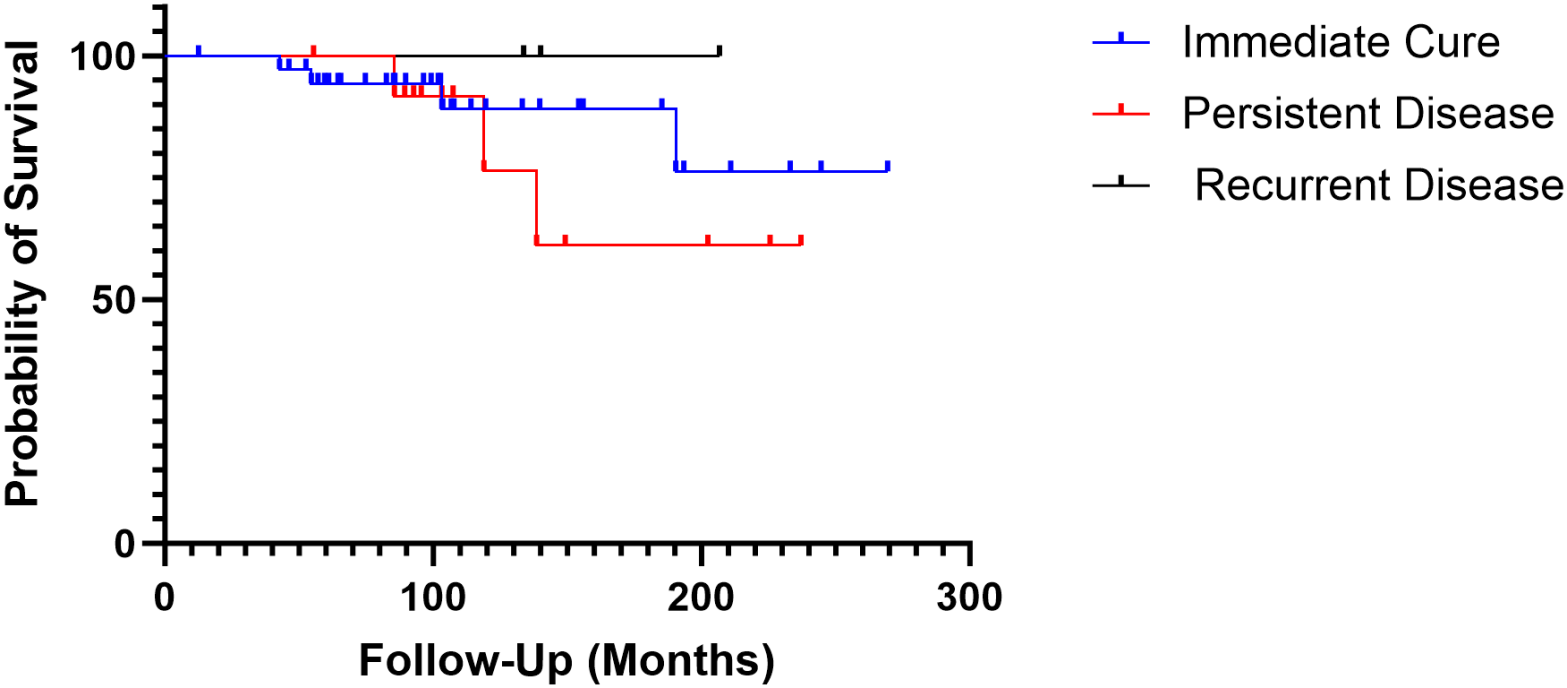
Kaplan–Meier curve showing cumulative probability of survival in NI CD patients stratified according to according to long-term cortisol status during follow-up. Log-rank *P* = 0.513.

There were seven deaths during the study (chronic renal disease *n* = 1, infection *n* = 2, malignancy *n* = 2, unknown *n* = 2). Median time to death from surgery was 10.1 (6.3–11.5) years. Standardised mortality ratio was significantly higher in the CD cohort than in the general population of NI (Table 2). There was no significant difference in survival between the remission, recurrent and persistent disease groups (Fig. 2).

**Table 2:** Standardised mortality ratios (SMR) in CD diagnosed in NI 2000–2019 compared to age-, sex- and calendar year-specific rates for the NI population. Fisher’s exact test was used to calculate CIs and significance.

	Observed	Expected	Standardised mortality ratio	95% CI	*P*
Overall	7	0.86	8.10	3.3–16.7	< 0.001
Long-term remission	4	0.66	6.05	1.65–15.5	0.005
Persistent disease	3	0.20	14.8	3.0–43.1	0.001

In the eight patients who proceeded to bilateral adrenalectomy, two had radiological and biochemical evidence of corticotroph tumour progression syndrome after bilateral adrenalectomy as defined by recent consensus (Reincke *et al.* 2021).

One case in the cohort met criteria for an aggressive pituitary neuroendocrine tumour (Raverot *et al.* 2018). This was a case previously reported associated with a pathogenic constitutional *MSH2* variant (Loughrey *et al.* 2021). Following this report the patient had limited response to nivolumab anti-PD-1 immunotherapy with stable tumour residuum and rising ACTH levels, although the patient was hypercortisolaemic during the immunotherapy. She also did not respond to pasireotide and is currently being considered for repeat radiotherapy.

## Discussion

This retrospective study describes a population-representative surgical CD cohort in NI diagnosed over a period of 20 years. These novel data reinforce CD as a rare disease, with an estimated incidence of 1.39–1.57 per million population per year in NI. This annual incidence is consistent with other Northern European cohorts reporting annual incidences of approximately 1.2–1.7 cases per million population per year (Lindholm *et al.* 2001, Agustsson *et al.* 2015, Ragnarsson *et al.* 2019, Wengander *et al.* 2019). Elsewhere in the world CD annual incidence has been reported at 1.8 cases per million population per year (Bolland *et al.* 2011). A neurosurgical cohort of CD has previously been reported in NI, thus providing an opportunity to draw comparisons between CD data in 2000–2019 and1979–2000 (Atkinson *et al.* 2005). In that study, 63 patients undergoing a first pituitary surgery for CD were analysed. Considering an overlap of 1 year between this study and Atkinson and colleagues’ report (year 2000, one patient), there was a decrease in the average number of patients operated from 2.86 per year to 2.74 per year in the present study. Over the last 40 years the data indicate there has been no apparent increase in annual rates of CD diagnosis in NI despite the population increasing by approximately 13% (NISRA 2021). This may reflect a true decrease in the incidence of CD in NI, although consideration must be given to the possibility that patients from NI were operated elsewhere or that individuals from NI may have relocated and developed disease while living elsewhere. It may also suggest that the condition remains under-diagnosed.

Comorbidities were prevalent in this study, highlighting the multi-system burden of CD. Hypokalaemia was detected in approximately one-third of the current CD cohort on at least one occasion prior to diagnosis, and the presence of hypokalaemia was significantly associated with higher 24-h UFC. Hypokalaemia has previously been reported in other CD populations, but at a lower frequency (6–26%) than in the current study (Hassan-Smith *et al.* 2012, Fan *et al.* 2020). One consideration is the possibility of medications contributing to hypokalaemia, but unfortunately, sufficient data on medications contributing to hypokalaemia in this NI cohort are not available.

Almost one in ten patients had received a diagnosis of VTE at the point of study close and this is a comorbidity which is now well recognised as a complication of CD (Wagner *et al.* 2018, Isand *et al.* 2024). Patients with CD in NI are not routinely screened for VTE; therefore, an underestimation of VTE in this cohort is possible. Although recent CD guidelines suggest low-molecular-weight heparin as a thromboprophylaxis option, no formal CD thromboprophylaxis guidance exists (Fleseriu *et al.* 2021, Isand *et al.* 2024).

Osteoporosis and low-impact fractures are a similar challenge in this group of patients with CD and this is evidenced in the large European Register of Cushing’s Syndrome cohort (Valassi *et al.* 2011). Recent CD guidelines advise that dual X-ray absorptiometry may be insufficient to detect osteoporosis in CD and that consideration should be given to treatment with bisphosphonates in those patients with persistent CD (Fleseriu *et al.* 2021). Despite the burden of symptoms and comorbidities in this NI cohort, the diagnostic delay often experienced by patients with CD was once again evident (Valassi *et al.* 2017).

The data in this study indicate that pituitary surgery in NI is safe and continues to show comparably good outcomes relative to other larger centres. Metyrapone was used as preoperative therapy in 34% of the cohort, and this is a higher proportion than that reported in the European Register of Cushing’s Syndrome cohort (20%) (Valassi *et al.* 2018). This is most likely due to familiarity with metyrapone in NI and alternative use of ketoconazole in the European Register of Cushing’s Syndrome cohort (Valassi *et al.* 2018). Sixteen percent of patients had a simple cerebrospinal fluid leak that was repaired intraoperatively, and five patients had a persistent postoperative cerebrospinal fluid leak (one requiring surgical repair). These rates are comparable to other neurosurgical centres in the UK and Ireland (Hassan-Smith *et al.* 2012, Brady *et al.* 2021). Similarly, rates of transient (22%) and permanent AVP-D (19%) observed in this study are in line with other recent CD cohorts, with reported rates ranging from 33% to 35% and from 3% to 23% respectively Hassan-Smith *et al.* 2012, Saini *et al.* 2019, Brady *et al.* 2021). A 2015 meta-analysis calculated a rate of postoperative AVP-D of 9% (range: 0–75), which is once again comparable to the current study. In this NI CD cohort, the data are not complete, but it is estimated that the majority (minimum 49/53 (92%)) of patients were operated via a microscopic trans-sphenoidal approach. Rates of AVP-D are almost certainly related to the surgical technique. In general, the neurosurgical approach in NI has been to achieve remission with a reasonably radical resection. This is particularly so in cases with no discrete tumour identified radiologically or intraoperatively or when attempting to avoid multiple surgeries in frail, comorbid patients. In these instances, a subtotal excision of the gland was undertaken, carefully considering the balance of risks and benefits of CD remission versus permanent AVP-D.

Interestingly, in this Northern Irish cohort, the identification of a corticotrophinoma in histopathological analysis did not seem to confer an increased likelihood of immediate postoperative remission or indeed longer-term remission from a single surgery. Other literature has suggested that consideration should be given to early reoperation if a corticotrophinoma is not identified on histopathological analysis (Ram *et al.* 1994, Asuzu *et al.* 2017). However, data from the same centre suggest that a lack of identifiable corticotrophinoma on histopathological analysis is not associated with early remission in operated CD, and this is consistent with the current study (Asuzu *et al.* 2017). Furthermore, a meta-analysis of CD pituitary surgery outcomes found that identification of adenomatous tissue was predictive of remission (Stroud *et al.* 2020). Like postoperative AVP-D, presence of tumour tissue on histopathological analysis is affected by surgical technique and sampling. This may explain the variable findings in the literature. Suspicion of non-remission if a corticotrophinoma is not identified is wise; however, it should not be the only consideration when deciding on the efficacy of a first surgery for CD.

When considering immediate remission in the current cohort, applying cut-offs of ≤ 50 nmol/L and ≤ 138 nmol/L resulted in early remission rates of 53% and 68% respectively. The reason for considering two thresholds is because various postoperative morning serum cortisol values have been described to define remission. In 2015 the Endocrine Society guidelines endorsed a cut-off of 138 nmol/L (5 µg/dL), 2021 guidelines discussed a threshold of ≤ 55 nmol/L (2 µg/dL) and in Europe it has been convention to use a cut-off of 50 nmol/L (1.8 µg/dL) to define immediate postoperative remission of CD (Nieman *et al.* 2015, Fleseriu *et al.* 2021). These varying cut-offs and study heterogeneity can make a definition of remission and comparison between operated CD populations challenging. Reporting both thresholds in this study facilitates such comparisons. Other studies have quoted remission rates of 58–90% when using a strict 50–55 nmol/L threshold and 47–91% with a standard 138–140 nmol/L threshold (Lindsay *et al.* 2011, Hassan-Smith *et al.* 2012, Ironside *et al.* 2018, Saini *et al.* 2019, Brady *et al.* 2021, Catalino *et al.* 2023). Early postoperative CD remission rates in NI are therefore comparable to other countries with higher volume centres and reflects the importance of an experienced and skilled neurosurgeon in the management of CD.

Ultimately, while it is interesting to consider different cut-offs in immediate postoperative remission, there is no level of postoperative serum cortisol that definitively rules out the possibility of CD recurrence. What could be considered most important for the patient is the chance of obtaining longer-term remission from a single pituitary surgery. When comparing the long-term remission rate in the current study to that observed previously in NI, it appears to have improved over 40 years (Atkinson *et al.* 2005). Previously an overall remission rate of 56% was reported for the years 1979–2000 compared to 70% during the current study period 2000–2019 (Fig. 1) (Atkinson *et al.* 2005). Again, due to study heterogeneity it is difficult to draw comparisons to other cohorts, but more recent studies since 2010 appear to report long-term CD remission rates from a single pituitary surgery in the range 62–94% (Lindsay *et al.* 2011, Hassan-Smith *et al.* 2012, Yamada *et al.* 2012, Alexandraki *et al.* 2013, Catalino *et al.* 2023, Mondin *et al.* 2023).

There is a population of patients with CD in NI who appear to not achieve strict or standard immediate remission thresholds but who go on to achieve long-term remission without further intervention (*n* = 12, 23% and *n* = 6, 11% respectively). Similar instances have been reported in other cohorts (Lindsay *et al.* 2011, Saini *et al.* 2019). It is difficult to account for this, but variable onset of postoperative hypocortisolaemia has been described (Ironside *et al.* 2018). Another consideration is that they had a milder degree of cortisol excess preoperatively resulting in less corticotroph suppression; however, the non-significant differences in degree of preoperative 24-h UFC elevation when compared to those who did achieve early remission with subsequent long-term remission suggest that this may not be the case.

The mortality associated with CD is once again demonstrated in the current study with a significantly elevated standardised mortality ratio in patients with CD (8.10) compared to the general population of NI. A recent meta-analysis of mortality in Cushing’s syndrome reported an overall SMR in CD of 2.8 (range: 1.42–9.30), and the results of the current study are consistent with this (Limumpornpetch *et al.* 2022). The meta-analysis also indicated a significant difference in SMRs between active CD and remission, and a similar difference has been observed in the SMRs of the current cohort (Table 2); however, this is not evidenced in the Kaplan–Meier curves (Fig. 2). Notably, the three patients with a disease recurrence have 100% survival, and this is likely due to small sample size and follow-up duration (a period of increased Cushing’s syndrome mortality has been described in the time > 10 years’ follow-up) (Limumpornpetch *et al.* 2022). Survival differences when compared to other studies may also be influenced by differences in study designs.

The obvious limitation of this study is its retrospective nature. This is particularly relevant to documentation of symptoms and their duration. Patients were seen by a number of different clinicians; therefore, history taking and documentation would have been variable. This could introduce bias. The cohort is also relatively small compared to other multinational cohorts and reports from centres serving larger populations. Furthermore, the follow-up duration is modest, particularly when considering the late recurrences and mortality that can be seen in CD. This limits the analyses that can be undertaken and the subsequent conclusions that can be drawn.

Despite the small population size, the cohort has been meticulously characterised and is a population-representative one of the whole of NI across a 20-year period. The data (including available MRI interpretation) were collated by a single individual (PBL) from a single centre and therefore inter-observer effect on data collection is eliminated. The service has maintained subspecialised pituitary surgery with one to two pituitary neurosurgeons across the 20-year study period. Finally, given the importance of CD as a rare entity, the data have been presented in such a way to be consistent with other cohorts to facilitate comparison and future meta-analyses.

## Conclusion

This study provides novel insights on epidemiology and mortality in a population-based cohort of patients with CD in NI. CD remains a rare disease in NI, and despite a rising population, the number of patients annually diagnosed with CD has not increased. Trans-sphenoidal pituitary surgery is safe in NI, and a single surgery can achieve long-term remission in approximately 70% of cases. The median time to recurrence reinforces the importance of lifelong follow-up for patients with CD. Despite the benefits of surgery, patients with CD are vulnerable to multiple comorbidities and have an increased mortality compared to the general population of NI.

## Declaration of interest

MK is a Deputy Editor of *Endocrine-Related Cancer*. MK was not involved in the review or editorial process of this paper, on which she is listed as an author. The other authors declare that there is no conflict of interest that could be perceived as prejudicing the impartiality of the study reported.

## Funding

This work was supported by HSC R&D Division, Public Health Agencyhttp://dx.doi.org/10.13039/501100001626 (EAT/5498/18).

## Author contribution statement

LPB: conceptualization, data curation, formal analysis, investigation, methodology, project administration, visualization, writing – original draft, writing – review and editing; HB: data curation, writing – review and editing; CS: data curation, writing – review and editing; WP: writing – review and editing; SJE: data curation, writing – review and editing; MKR: writing – review and editing; HEG: writing – review and editing; EJ: data curation, writing – review and editing; CSG: methodology, supervision, writing – review and editing; JJA: conceptualization, methodology, resources, supervision, writing – review and editing; KM: methodology, supervision, writing – review and editing; HSJ: supervision, writing – review and editing.
